# Steroids in the Management of Ionotropic-Resistant Septic Shock: A Comprehensive Review of Efficacy and Outcomes

**DOI:** 10.7759/cureus.67795

**Published:** 2024-08-26

**Authors:** Nimmanagoti Nagaraju, Ashish Varma, Revat J Meshram

**Affiliations:** 1 Paediatrics, Jawaharlal Nehru Medical College, Datta Meghe Institute of Higher Education and Research, Wardha, IND

**Keywords:** mortality reduction, hemodynamic stability, hydrocortisone, steroid therapy, ionotropic resistance, septic shock

## Abstract

Septic shock is a critical condition characterized by persistent hypotension despite adequate fluid resuscitation and the use of vasopressors, often accompanied by multi-organ dysfunction. A challenging subset, ionotropic-resistant septic shock, persists despite ionotropic support. Steroids have been explored as a treatment for septic shock due to their anti-inflammatory properties and potential to improve hemodynamic stability. This review aims to evaluate the efficacy and outcomes of steroid therapy in managing ionotropic-resistant septic shock, assessing its impact on mortality, hemodynamic parameters, and adverse effects. A comprehensive review of the current literature, including randomized controlled trials, observational studies, and clinical guidelines, was conducted. Key studies, such as the CORTICUS and ADRENAL trials, were analyzed to determine the effectiveness of steroid regimens, specifically low-dose hydrocortisone, in patients with septic shock resistant to ionotropic agents. Evidence from recent trials indicates that low-dose hydrocortisone therapy can improve hemodynamic stability and reduce mortality in patients with septic shock, including those with ionotropic resistance. However, the benefits may vary depending on the timing of intervention, patient characteristics, and the presence of contraindications. Steroid therapy is associated with potential adverse effects, including secondary infections, glucose dysregulation, and gastrointestinal issues. Steroid therapy, particularly low-dose hydrocortisone, appears to be an effective adjunctive treatment for ionotropic-resistant septic shock, offering improved shock reversal and reduced mortality. Nonetheless, careful consideration of the risks and benefits is essential, and ongoing research is needed to refine treatment protocols and optimize patient outcomes. This review provides a detailed synthesis of current evidence and offers recommendations for clinical practice and future research in the management of septic shock resistant to ionotropic agents.

## Introduction and background

Septic shock is a severe manifestation of sepsis, defined by persistent hypotension despite adequate fluid resuscitation and the necessity for vasopressors to maintain mean arterial pressure (MAP) at a normal level [1]. This critical condition represents a progression from sepsis, an overwhelming systemic response to infection that leads to widespread inflammation, vasodilation, and multi-organ dysfunction. The underlying pathophysiology of septic shock involves a complex interaction between the host's immune response and the infecting pathogen [2]. The initial infection triggers a systemic inflammatory response syndrome (SIRS), resulting in the release of pro-inflammatory cytokines and mediators. This cascade leads to vasodilation, increased vascular permeability, and impaired cardiac contractility, ultimately resulting in reduced tissue perfusion and oxygen delivery. The persistence of hypotension and multi-organ failure marks a significant increase in morbidity and mortality rates in septic shock patients [2]. A specific subset of septic shock, known as ionotropic-resistant septic shock, is characterized by inadequate cardiovascular response to ionotropic agents, which are typically used to enhance cardiac contractility and improve hemodynamic status [3]. This condition complicates the management of septic shock, as patients continue to experience hypotension despite optimal fluid resuscitation and the use of ionotropic and vasopressor therapies. The continued presence of shock symptoms, despite these interventions, highlights the severity of the underlying pathophysiological alterations, such as profound myocardial depression and dysregulation of systemic vascular resistance [4].

The use of steroids in the management of sepsis has a long history. The introduction of steroids as anti-inflammatory agents began in the early 20th century, with initial studies suggesting potential benefits in reducing inflammation and modulating immune responses in septic patients. However, early enthusiasm was tempered by concerns over safety and efficacy, particularly due to the risk of secondary infections and other adverse effects [5]. Over recent decades, the role of steroids in sepsis management has been revisited with more robust clinical evidence. Landmark trials, such as the CORTICUS and ADRENAL studies, have demonstrated that low-dose hydrocortisone can be beneficial in reducing mortality and improving hemodynamic stability in patients with septic shock, including those with ionotropic resistance [6]. Current guidelines reflect this updated understanding of steroid therapy. Major health organizations, including the Surviving Sepsis Campaign, now recommend the use of low-dose hydrocortisone (200 mg/day) for patients with septic shock who remain hypotensive despite adequate fluid resuscitation and vasopressor therapy [7]. This recommendation is based on evidence indicating that steroid therapy can enhance shock reversal and reduce mortality, although the benefits may vary depending on patient characteristics and the timing of intervention. Despite these guidelines, ongoing debate and research continue regarding the optimal dosing, timing, and duration of steroid therapy, as well as its potential risks [8].

The purpose of this review is to provide a comprehensive evaluation of the efficacy and outcomes associated with steroid use in managing ionotropic-resistant septic shock. By synthesizing current evidence from clinical trials, observational studies, and guideline recommendations, this review seeks to clarify the role of steroids in this challenging clinical scenario. It will explore the mechanisms through which steroids exert their effects, assess the benefits and risks, and provide insights into current practice guidelines and future research directions. Ultimately, this review aims to enhance the understanding of how to optimize steroid therapy in the treatment of patients with ionotropic-resistant septic shock and inform clinical decision-making.

## Review

Mechanism of action of steroids in septic shock

Corticosteroids are crucial in managing septic shock, primarily due to their anti-inflammatory and hemodynamic effects. Understanding these mechanisms is essential for optimizing treatment strategies in patients who do not respond to standard therapies [9]. One of the primary mechanisms by which corticosteroids exert their effects is through their anti-inflammatory properties. They inhibit the nuclear factor-kappa B (NF-κB) pathway, a key regulator of the inflammatory response. This inhibition results in reduced production of pro-inflammatory cytokines, including interleukin (IL)-1, IL-6, and tumor necrosis factor-alpha (TNF-α) [10]. By modulating the immune response, corticosteroids help alleviate the hyper-inflammatory state characteristic of septic shock, which can cause excessive vasodilation and hypotension, complicating the clinical picture. Additionally, corticosteroids decrease the release of other inflammatory mediators, such as nitric oxide (NO), by inhibiting nitric oxide synthase. This action is particularly beneficial in counteracting sepsis-induced vasodilation, thereby improving vascular tone and blood pressure [11]. Beyond their anti-inflammatory effects, corticosteroids provide significant hemodynamic benefits. They enhance vascular tone by facilitating the binding of catecholamines to beta-adrenergic receptors, thereby improving the responsiveness of blood vessels to vasopressors. This interaction is critical in septic shock, where severe hypotension occurs due to widespread vasodilation [12]. By improving vascular responsiveness, corticosteroids help stabilize blood pressure and enhance perfusion to vital organs, which is crucial for patient recovery. The hemodynamic benefits of corticosteroids are also attributed to their ability to increase adrenergic receptor sensitivity. In septic shock, the body’s response to catecholamines may be diminished, making it challenging to maintain adequate blood pressure control. By enhancing adrenergic receptor sensitivity, corticosteroids increase the effectiveness of vasopressors used to treat hypotension, thereby contributing to better hemodynamic stability [13]. Table 1 summarizes the mechanism of action of steroids in septic shock.

**Table 1:** Summarizing the mechanism of action of steroids in septic shock.

Mechanism	Description	Effect
Inhibition of nuclear factor-kappa B (NF-κB) pathway	Corticosteroids inhibit the NF-κB pathway, a key regulator of the inflammatory response.	Reduces production of pro-inflammatory cytokines like IL-1, IL-6, and tumor necrosis factor-alpha (TNF-α), mitigating inflammation.
Reduction of inflammatory mediators	Corticosteroids decrease the release of inflammatory mediators such as nitric oxide (NO) by inhibiting nitric oxide synthase.	Counteracts sepsis-induced vasodilation, leading to improved vascular tone and blood pressure.
Enhancement of catecholamine binding	Steroids facilitate the binding of catecholamines to beta-adrenergic receptors.	Improves responsiveness of blood vessels to vasopressors, stabilizing blood pressure.
Increase in adrenergic receptor sensitivity	Corticosteroids increase the sensitivity of adrenergic receptors.	Enhances the effectiveness of vasopressors in treating hypotension, improving hemodynamic stability.

Efficacy of steroids in ionotropic-resistant septic shock

Evidence From Clinical Trials

The efficacy of corticosteroids in managing inotropic-resistant septic shock has been evaluated in several key clinical trials, including the CORTICUS and ADRENAL studies. These trials provide valuable insights into the effectiveness of various steroid regimens, such as hydrocortisone, dexamethasone, and prednisone [13]. The CORTICUS trial aimed to assess the efficacy and safety of low-dose hydrocortisone in patients with septic shock. This multicenter, randomized, double-blind, placebo-controlled trial included 499 patients across 52 intensive care units. Participants received 50 mg of hydrocortisone every six hours for five days, followed by a tapering regimen. The trial found no significant difference in 28-day mortality between the hydrocortisone group (39.2%) and the placebo group (36.1%) among patients who did not respond to a corticotropin test. However, hydrocortisone treatment was associated with quicker shock reversal, although it also led to a higher incidence of superinfections [14]. The ADRENAL trial sought to determine the effects of hydrocortisone on mortality in patients with septic shock. This trial involved over 3,800 patients and compared hydrocortisone (200 mg/day) with placebo over a seven-day period. The results indicated that hydrocortisone did not significantly reduce mortality at 90 days compared to placebo. However, it did improve shock reversal and was associated with fewer days on mechanical ventilation, highlighting its potential benefits in managing septic shock [15]. In addition to these pivotal trials, several meta-analyses have evaluated the combined data from various studies. These analyses suggest that prolonged low-dose corticosteroid treatment (200-300 mg/day) may reduce mortality and improve outcomes in septic shock patients, particularly those who are vasopressor-dependent. This evidence supports the use of corticosteroids in specific patient populations, although the optimal dosing and duration of treatment remain subjects of ongoing research [13]. When comparing steroid regimens, hydrocortisone is the most commonly used corticosteroid in septic shock management, typically administered at doses of 200-300 mg/day in divided doses. Studies indicate that hydrocortisone may improve shock reversal but does not consistently reduce mortality in septic shock patients. Dexamethasone, while effective in other contexts, is less frequently used in septic shock, and its role remains under investigation. Prednisone, on the other hand, is generally not recommended for acute septic shock due to its longer half-life and delayed onset of action compared to hydrocortisone [16].

Subgroup Analyses

Subgroup analyses of corticosteroid efficacy in inotropic-resistant septic shock have provided valuable insights into the effects of these treatments on different patient populations and their comparison with other treatment modalities, such as vasopressors. Recent studies have conducted subgroup analyses to evaluate the effects of corticosteroids based on various factors, including age, comorbidities, and the severity of sepsis [13]. For instance, a systematic review found no significant differences in the efficacy of corticosteroids among distinct clinical subgroups, including patients with septic shock, pneumonia, or acute respiratory distress syndrome (ARDS). The evidence suggests that while corticosteroids may reduce mortality overall, this effect does not vary meaningfully across different patient populations. However, the absolute reduction in mortality is likely greater in patients with a higher baseline risk of death, indicating that sicker patients may derive more benefit from corticosteroid treatment [17]. Additionally, subgroup analyses have compared the efficacy of different corticosteroids, such as hydrocortisone, methylprednisolone, and dexamethasone, finding no substantial differences among these agents. The absence of a credible effect modification based on patient characteristics suggests that corticosteroids can be broadly applied across various patient populations with septic shock, though the risks of adverse effects, such as hyperglycemia and neuromuscular weakness, may be more pronounced in sicker patients [18]. When comparing corticosteroids to other treatment modalities, particularly vasopressors, evidence indicates that corticosteroids may enhance the effectiveness of vasopressor therapy. The Surviving Sepsis Campaign (SSC) guidelines recommend using corticosteroids in adult patients with septic shock who require ongoing vasopressor support. The combination of corticosteroids and vasopressors facilitates quicker shock reversal and may lead to improved hemodynamic stability, although the overall impact on mortality remains a subject of debate [4]. In clinical trials, such as the CORTICUS and ADRENAL studies, corticosteroids were shown to improve shock reversal rates; however, they did not consistently demonstrate a significant reduction in mortality compared to placebo. For example, the ADRENAL trial involved over 3,800 patients and found no significant difference in 90-day mortality between those receiving hydrocortisone and those receiving placebo, despite improvements in other clinical outcomes [19]. While corticosteroids may not provide a mortality benefit across various patient subgroups, they may still play a crucial role in managing septic shock, particularly in conjunction with vasopressor therapy. Current evidence supports their use in patients who do not respond adequately to fluid resuscitation and vasopressors, underscoring the need for individualized treatment approaches based on patient characteristics and clinical status. Further research, particularly individual patient data meta-analyses, may help clarify the nuances of corticosteroid efficacy in specific populations and treatment contexts [13].

Outcomes Associated With Steroid Use

Corticosteroids have become a significant research focus in managing septic shock, particularly regarding their impact on both short-term and long-term outcomes. In terms of short-term outcomes, the effects of corticosteroid use on mortality rates have yielded mixed results [13]. Some studies suggest that corticosteroids may reduce short-term mortality in septic shock patients, with a risk ratio of 0.93 (95% CI, 0.88-0.99), indicating a potential benefit. However, other research has shown an increase in 28-day mortality in specific populations, particularly those with gram-negative bacterial infections. These conflicting findings underscore the complexity of septic shock management and the need for careful consideration of corticosteroid use [13]. Beyond mortality rates, corticosteroids have been associated with improvements in hemodynamic stability over time. Patients receiving corticosteroid therapy often experience quicker shock reversal, typically within seven days, compared to those receiving standard care or placebo. This rapid response is crucial for effectively managing septic shock, especially in patients resistant to fluid resuscitation and vasopressor therapy. The ability to swiftly stabilize hemodynamics can significantly impact patient outcomes and potentially reduce the risk of further complications [20]. Regarding long-term outcomes, the influence of corticosteroids on ICU and hospital length of stay remains an area of ongoing investigation. While some studies suggest that corticosteroid therapy may reduce overall hospital stay, the evidence is inconclusive. This variability highlights the need for further research to establish a clearer understanding of how corticosteroids affect the duration of hospitalization in septic shock patients [21]. Corticosteroids also appear to impact organ function recovery in patients with septic shock positively. Evidence suggests that these steroids can enhance recovery rates, particularly in those with severe sepsis or septic shock. However, the degree of recovery can vary based on the severity of the illness and the timing of steroid administration. Overall, while corticosteroids can provide short-term mortality and hemodynamic stability benefits, their long-term effects on hospital stay and organ recovery require further investigation to establish definitive outcomes. The variability in study results underscores the importance of tailored approaches based on individual patient circumstances [22].

Safety and side effects

Adverse Effects of Steroids in Septic Shock

**Figure 1:**
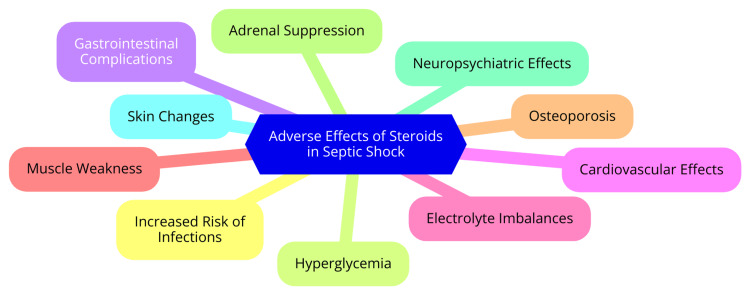
Adverse effects of steroids in septic shock. Image Credit: Nimmanagoti Nagaraju.

While corticosteroids can be beneficial in managing inotropic-resistant septic shock, they are not without risks. One of the primary concerns is the increased risk of secondary infections. Corticosteroids exert immunosuppressive effects, compromising the body’s ability to fight infections. This makes careful monitoring for signs of secondary infections essential, especially in patients receiving prolonged courses of steroids. The potential for infections can complicate the clinical picture and require additional interventions [13]. Another significant adverse effect of corticosteroids is their impact on glucose metabolism. These medications can induce hyperglycemia, worsening glycemic control in patients, particularly those with pre-existing diabetes or those who are already critically ill. Close monitoring of blood glucose levels is crucial, and prompt treatment of hyperglycemia is necessary to avoid complications such as diabetic ketoacidosis or hyperglycemic hyperosmolar state [23]. Additionally, corticosteroids may lead to gastrointestinal and cardiovascular complications. The risk of gastrointestinal bleeding is heightened, necessitating prophylactic measures in at-risk patients. Cardiovascular effects, such as hypertension and arrhythmias, can also occur, further complicating the management of septic shock. Therefore, patients receiving corticosteroids should be closely monitored for these potential side effects to ensure timely intervention [24]. Adverse effects of steroids in septic shock are shown in Figure 1.

Risk-Benefit Analysis

When considering corticosteroids in septic shock, balancing the potential benefits with the associated risks is crucial. The decision to use steroids should be individualized based on the patient's clinical status, severity of illness, and response to conventional therapies. Generally, corticosteroids are more likely to be beneficial in patients with severe, refractory septic shock who remain hypotensive despite adequate fluid resuscitation and high-dose vasopressor therapy. In these cases, corticosteroids' anti-inflammatory and vasopressor-sparing effects can be particularly advantageous [13]. Conversely, in patients with less severe shock or those who are responding well to conventional therapies, the risks associated with corticosteroids may outweigh the potential benefits. In such cases, a more conservative approach may be more appropriate, focusing on supportive care and minimizing the use of immunosuppressive agents [13]. Table 2 summarizes the safety and side effects of steroids in septic shock.

**Table 2:** Summarizing the safety and side effects of steroids in septic shock.

Category	Description	Potential impact
Secondary infections	Corticosteroids have immunosuppressive effects, increasing the risk of secondary infections.	Compromised immune response, making patients more susceptible to additional infections.
Glucose dysregulation	Steroids can induce hyperglycemia, particularly in critically ill patients or those with diabetes.	Worsened glycemic control, with risks of complications like diabetic ketoacidosis or hyperosmolar state.
Gastrointestinal effects	Increased risk of gastrointestinal bleeding, especially in patients with pre-existing conditions.	May require prophylactic measures; risk of peptic ulcers or GI hemorrhage.
Cardiovascular effects	Corticosteroids can cause hypertension, arrhythmias, and other cardiovascular complications.	Potential for worsening cardiovascular instability, necessitating close monitoring of blood pressure and heart rhythm.
Neuromuscular weakness	Prolonged corticosteroid use can lead to neuromuscular weakness, particularly in ICU patients.	Risk of prolonged mechanical ventilation and delayed recovery due to muscle weakness.

Current guidelines and recommendations

The management of sepsis and septic shock is guided by several international and national guidelines, with the Surviving Sepsis Campaign (SSC) and the National Institute for Health and Care Excellence (NICE) being among the most prominent. These guidelines provide a comprehensive framework for clinicians, ensuring timely and effective treatment for patients experiencing these critical conditions [25]. The SSC guidelines emphasize the importance of immediate treatment for suspected sepsis or septic shock. They recommend initiating fluid resuscitation with at least 30 mL/kg of intravenous crystalloid fluid within the first three hours for patients showing signs of sepsis-induced hypoperfusion. If hypotension persists despite adequate fluid resuscitation, the guidelines advocate for initiating vasopressor therapy to maintain a mean arterial pressure (MAP) of 65 mmHg or higher. Broad-spectrum antibiotics should also be administered immediately, ideally within the first hour of sepsis recognition. The guidelines stress the need for continuous monitoring and dynamic measures to guide fluid resuscitation rather than relying solely on static measures [26]. Recent updates to the SSC guidelines emphasize performance improvement programs within hospitals. The 2021 recommendations highlight the importance of screening for high-risk patients and implementing comprehensive assessment tools, moving away from reliance on the quick Sequential Organ Failure Assessment (qSOFA) as a standalone screening tool. Additionally, there is an increased focus on the long-term effects of sepsis, with recommendations for early and ongoing follow-up care to address physical rehabilitation and psychological support for survivors [27]. Similarly, the NICE guidelines offer a robust framework for managing sepsis in adults and children. They emphasize early recognition of sepsis symptoms and prompt initiation of treatment, aligning closely with the SSC recommendations. The guidelines advocate for administering antibiotics as soon as sepsis is suspected, alongside immediate fluid resuscitation and the use of vasopressors when necessary. NICE also strongly emphasizes educating healthcare professionals about recognizing sepsis and understanding management protocols [28]. Recent updates to the NICE guidelines reflect a shift toward patient-centered care. They underscore the importance of involving patients and their families in care decisions, mirroring the changes observed in the SSC guidelines. This approach enhances the quality of care and supports patients' and their families' emotional and psychological needs during a critical time [29]. Table 3 summarizes the current guidelines and recommendations for managing septic shock, including corticosteroids.

**Table 3:** Summary of the current guidelines and recommendations for managing septic shock, including corticosteroids.

Guideline/organization	Key recommendations	Details
Surviving Sepsis Campaign (SSC)	Use low-dose hydrocortisone (200 mg/day) in patients with septic shock who remain hypotensive despite adequate fluid resuscitation and vasopressor therapy.	- Immediate fluid resuscitation with 30 mL/kg of IV crystalloid within the first three hours. - Initiate vasopressors if mean arterial pressure < 65 mmHg despite fluids. - Administer broad-spectrum antibiotics within one hour.
National Institute for Health and Care Excellence (NICE)	Early recognition and prompt treatment of sepsis symptoms with antibiotics, fluid resuscitation, and vasopressors.	- Administer antibiotics as soon as sepsis is suspected. - Fluid resuscitation and vasopressor use when necessary. - Emphasis on educating healthcare professionals on sepsis recognition and management.
2021 SSC Update	Performance improvement programs for sepsis management within hospitals, emphasizing long-term follow-up care for survivors.	- Screening for high-risk patients and comprehensive assessment tools. - Focus on early follow-up care, physical rehabilitation, and psychological support.
Patient-Centered Care Emphasis (NICE)	Involve patients and families in care decisions, focusing on patient-centered approaches to improve the quality of care.	- Enhances communication and care quality by considering patient preferences and emotional needs.

Future directions and research needs

As the management of ionotropic-resistant septic shock continues to evolve, several key areas require further exploration to enhance patient outcomes. These areas include gaps in current knowledge regarding corticosteroid use and emerging therapies that could shape future research [30]. One significant gap in current knowledge is the need for further research on the optimal dosing and timing of corticosteroid administration. While existing guidelines recommend low-dose hydrocortisone for septic shock, there remains uncertainty about the best dosing strategies. Future studies should focus on defining optimal dosages, including the potential benefits of high-dose versus low-dose regimens. Additionally, the timing of corticosteroid initiation is crucial; research should investigate the effects of early versus late administration on patient outcomes, particularly in different subgroups of septic shock patients. This information could help clinicians make more informed decisions that improve survival rates and recovery times [13]. Another area that warrants exploration is the investigation of alternative steroid regimens. Current research predominantly focuses on hydrocortisone, but there is a need to evaluate other corticosteroids, such as dexamethasone or prednisone, in septic shock. Furthermore, the potential benefits of combination therapies that pair corticosteroids with other agents should be studied to enhance therapeutic effects while minimizing side effects. This exploration could lead to more effective treatment protocols tailored to individual patient needs [13]. In addition to corticosteroids, there is a growing interest in adjunctive therapies that could complement standard treatment protocols. Investigating novel anti-inflammatory agents, such as interleukin inhibitors or monoclonal antibodies, may provide insights into modulating the inflammatory response in septic shock. Furthermore, exploring immunomodulatory therapies that enhance immune function or restore immune balance in septic patients could lead to new treatment options that improve outcomes [31]. Finally, the potential for personalized medicine approaches represents a promising frontier in managing septic shock. Research should focus on identifying biomarkers that predict patient responses to corticosteroids and other therapies, allowing for more tailored treatment strategies based on individual profiles. Understanding how genetic variations influence treatment responses could also lead to more effective and individualized therapeutic approaches, ultimately improving patient care [32]. Table 4 summarizes the future directions and research needs in managing ionotropic-resistant septic shock with a focus on corticosteroid use.

**Table 4:** Summary of the future directions and research needs in managing ionotropic-resistant septic shock with a focus on corticosteroid use.

Area of research	Research needs	Potential impact
Optimal dosing and timing of corticosteroids	Further research on the best dosing strategies, including high-dose vs. low-dose regimens and the timing of corticosteroid initiation.	Improved patient outcomes through more precise dosing protocols tailored to the severity and timing of septic shock.
Alternative steroid regimens	Evaluation of other corticosteroids like dexamethasone or prednisone, and potential combination therapies with other agents.	Development of more effective and tailored treatment protocols that minimize side effects and improve efficacy.
Adjunctive therapies	Investigation of novel anti-inflammatory agents (e.g., interleukin inhibitors and monoclonal antibodies) and immunomodulatory therapies.	Expansion of treatment options that could complement or replace corticosteroids, leading to better patient outcomes.
Personalized medicine approaches	Identification of biomarkers and genetic variations that predict patient responses to corticosteroids and other therapies.	Tailored treatment strategies based on individual patient profiles, potentially improving survival and recovery rates.
Long-term outcomes and survivorship	Research into the long-term effects of corticosteroid use in septic shock survivors, including impacts on quality of life and chronic health conditions.	Enhanced understanding of how corticosteroids affect long-term recovery, leading to improved follow-up care strategies.

## Conclusions

In conclusion, the use of steroids in managing ionotropic-resistant septic shock represents a significant advancement in the treatment of this severe condition. Current evidence supports the efficacy of low-dose hydrocortisone in improving hemodynamic stability and reducing mortality among patients with septic shock who remain hypotensive despite optimal fluid and vasopressor therapy. The mechanisms by which steroids exert their beneficial effects, including modulation of the inflammatory response and enhancement of cardiovascular function, are well-documented. However, the variability in patient responses, the potential for adverse effects, and the ongoing debate about optimal dosing and duration underscore the need for continued research. Adherence to updated clinical guidelines is crucial, but further studies are necessary to refine treatment protocols and address existing gaps in knowledge. Ultimately, a nuanced understanding of steroid therapy's role in septic shock management will contribute to improved patient outcomes and guide future therapeutic strategies.
